# Novel neoplasms associated with syndromic pediatric medulloblastoma: integrated pathway delineation for personalized therapy

**DOI:** 10.1186/s12964-022-00930-3

**Published:** 2022-08-17

**Authors:** Maria-Magdalena Georgescu, Stephen G. Whipple, Christina M. Notarianni

**Affiliations:** 1 NeuroMarkers PLLC, Houston, TX 77025 USA; 2 Department of Neurosurgery, Louisiana State University Shreveport, Shreveport, LA 71103 USA

**Keywords:** Medulloblastoma, Li-Fraumeni syndrome, Familial adenomatous polyposis, Atypical pituitary adenoma prolactinoma, Desmoid fibromatosis, Radiotherapy secondary malignancies, PARP inhibitors, Receptor tyrosine kinase (RTK) inhibitors, Prolactin receptor (PRLR) inhibitors

## Abstract

Medulloblastoma is the most common pediatric embryonal brain tumor, and may occur in cancer predisposition syndromes. We describe novel associations of medulloblastoma with atypical prolactinoma and dural high-grade sarcoma in Li-Fraumeni syndrome (LFS), and epidural desmoid fibromatosis in familial adenomatous polyposis (FAP)/Turcot syndrome. Genomic analysis showing *XRCC3* alterations suggested radiotherapy as contributing factor to the progression of LFS-associated medulloblastoma, and demonstrated different mechanisms of *APC* inactivation in the FAP-associated tumors. The integrated genomic-transcriptomic analysis uncovered the growth pathways driving tumorigenesis, including the prolactin-prolactin receptor (PRLR) autocrine loop and Shh pathway in the LFS-associated prolactinoma and medulloblastoma, respectively, the Wnt pathway in both FAP-associated neoplasms, and the TGFβ and Hippo pathways in the soft tissue tumors, regardless of germline predisposition. In addition, the comparative analysis of paired syndromic neoplasms revealed several growth pathways susceptible to therapeutic intervention by PARP, PRLR, and selective receptor tyrosine kinase (RTK) inhibitors. These could target the defective DNA damage repair in the LFS-associated medulloblastoma, the prolactin autocrine loop in the atypical prolactinoma, the *EPHA3/7* and *ALK* overexpression in the FAP-associated medulloblastoma, and the multi-RTK upregulation in the soft tissue neoplasms. This study presents the spatiotemporal evolution of novel neoplastic associations in syndromic medulloblastoma, and discusses the post-radiotherapy risk for secondary malignancies in syndromic pediatric patients, with important implications for the biology, diagnosis, and therapy of these tumors.

**Video Abstract**

**Supplementary Information:**

The online version contains supplementary material available at 10.1186/s12964-022-00930-3.

## Background

Medulloblastoma (MB) is a World Health Organization (WHO) grade 4 (highest grade) embryonal neoplasm, and accounts for 25% of all pediatric intracranial tumors [1]. Depending on the genomic alterations, MB is grouped into three molecular subgroups: MB-WNT, MB-SHH, and MB-nonWNT/nonSHH.

The MB-WNT subgroup accounts for approximately 10% of MBs, and contains tumors with mutations activating the Wingless (Wnt) signal transduction pathway (Additional file 1: Fig. S1). These are usually somatic activating mutations in *CTNNB1*, encoding β-catenin, in nearly 90% of cases. The remaining cases may harbor a germline inactivating alteration in *APC*, the tumor suppressor gene responsible for familial adenomatous polyposis (FAP) syndrome [2]. The prognosis of children with MB-WNT tumors is the best from all molecular subgroups, with overall 95% 5-year patient survival [3].

The MB-SHH subgroup accounts for 30% of MBs, but shows a skewed age distribution, with preponderance in two thirds of the adult and infant MB cases, but restricted to only 15% of the pediatric patients of age 3–16 years [4]. It contains tumors with alterations in many genes encoding mediators of the Sonic hedgehog (Shh) signaling pathway (Additional file 1: Fig. S1), such as *PTCH1* (43% of patients), *SUFU* (10%), *SMO* (9%), *GLI1* or *GLI2* (9%) and *MYCN* (7%) [2]. *TP53* mutations, if not associated with Wnt pathway gene mutations, assign the tumors to a separate, very high-risk subgroup of MB-SHH: MB-SHH/TP53-mutant, which roughly accounts for 20% of the MB-SHH cases [5]. MBs with *TP53* mutations almost exclusively occur in children of age 5–17 years, and induce only in the MB-SHH/TP53-mutant subgroup a very poor, 41% 5-year overall survival. Germline *TP53* mutations, the hallmark of Li-Fraumeni syndrome (LFS) [6], are also restricted to the MB-SHH/TP53-mutant subgroup and account for over half of these tumors [5]. Overall, *TP53* germline mutations are present 20% of MB-SHH tumors from children age 5–16 years, and confer a dismal 27% 5-year overall survival [7]. This prognosis is the worst of all MB subgroups, at par only with MB-nonWNT/nonSHH with *MYC* amplification [2]. Not all MB-SHH/TP53-mutant cases show Shh pathway mutations, but most show amplification of *MYCN*, *GLI2* or *SHH* and no alterations in *PTCH1*, in contrast to most of the other MB-SHH/TP53-wild-type cases [8]. These gene amplifications may be triggered by the presence of genome instability or chromothripsis in MBs-SHH/TP53-mutant [9].

LFS is not the only syndrome predisposing to MB [10]. In fact, consensus MB management recommends germline screening for all patients with MB-WNT lacking somatic *CTNNB1* mutations and for all patients with MB-SHH [7]. Beside *TP53*, MB-SHH may display germline mutations mainly in *PTCH1* or *SUFU* associated with Gorlin syndrome, but also germline variants in the DNA-damage response (DDR) genes *BRCA2* and *PALB2* [3, 7]. In addition to germline testing, the risk for associated malignancies and their relationship to MB treatment must be evaluated in the management of patients with MB [2].

In this study, we extend the spectrum of syndromic MB-associated neoplasms by exploring the temporal evolution and integrated pathway analysis of novel MB-associated malignancies in two pediatric patients with FAP and LFS cancer predisposition syndromes. Occurrence of cranial desmoid fibromatosis, a novel MB secondary malignancy, followed radiotherapy for MB in the FAP patient. In contrast, the LFS patient developed first recurrent atypical prolactinoma, a new malignancy for LFS, followed by MB progressing immediately after radiotherapy for prolactinoma, followed by dural high-grade sarcoma, a third malignancy arising yet in this patient in a relatively short span. The pathways leading to tumor progression were delineated, and new avenues for treatment of these tumors were highlighted.

## Methods

### Tumor specimens, histology and immunohistochemistry (IHC)

Surgical resection specimens were obtained from patients, as previously described, in accordance to hospital regulations [11–13]. Formalin-fixed paraffin-embedded (FFPE) sections were stained with hematoxylin–eosin (H&E). Images were acquired with Nikon Eclipse Ci microscope equipped with Nikon Digital Sight DS-Fi2 camera (Nikon Instruments Inc., Melville, NY), as previously described [14, 15]. IHC was performed on selected sections, by using the heat-induced epitope retrieval method at 95 °C in CC1 solution for 8 min on a Ventana Benchmark Ultra platform (Roche/Ventana Medical Systems Inc., Tucson, AZ), as previously described [12, 15]. The following primary antibodies and corresponding dilutions were used: NHERF1 1:2000 (Thermo/Fisher, Waltham, MA), beta-catenin 1:100, Prolactin (Rabbit polyclonal) 1:400, ACTH (Rabbit Polyclonal) 1:400, GH (Rabbit Polyclonal) 1:400, FSH (Rabbit Polyclonal) 1:50, LH (Rabbit Polyclonal) 1:400, GFAP (EP672Y) 1:100, Olig-2 (387 M-15) 1:50, h-caldesmon 1:50, CD68 (KP1) 1:400; SMA (1A4) 1:400; CD163 1:50, Myogenin (EP162) 1:100, EMA (E29) 1:400 (Ventana/ Cell Marque, Rocklin, CA), p53 (DO-7) 1:400, Synaptophysin (SP11) 1:250, Cytokeratin (CAM5.2) 1:100, Estrogen receptor (SP1) 1:200, Desmin (DE-R-11) 1:100, S100 (Rabbit Polyclonal) 1:400, Ki-67 (30–9) 1:200 (Roche/Ventana), GAB1 1:400 (H7) and YAP (63.7) 1:50 (Santa Cruz Biotechnology, Dallas, TX).

### Next generation sequencing (NGS), copy number (CN) variation, and transcriptomics

NGS and CN analysis were performed from FFPE samples by using the xT-596-gene or xT-648-gene panels (Tempus Labs, Chicago, IL), as previously described [11, 13, 16]. The ClinVar Database (https://www.ncbi.nlm.nih.gov/clinvar/) was accessed for the analysis of germ-line mutations. Clonal allele fraction was approximated at half of the tumor content percent (%) for heterozygous mutations, and approaching the tumor content % for mutations showing loss of heterozygosity (LOH). Gene amplification was called for CN ≥ 7. The tumor mutation burden (TMB) is expressed as single-nucleotide protein-altering mutations per megabase DNA. Whole transcriptome RNA sequencing with RNA fusion detection was performed at Tempus Labs for all samples from FFPE sections [11, 14]. For each case, the same FFPE block was used for DNA and RNA extraction to allow direct comparison of results. The expression analysis and functional gene classification were performed as described [11]. Briefly, the threshold for total RNA counts was set at ≥ 500 in at least one tumor sample, and pseudogenes and Y-chromosome genes were excluded. A ≥ 5-fold overexpression threshold was set relative to lowest values from one of the syndromic tumors or from MB-SHH tumor control (CTR1). Absolute expression levels of selected overexpressed genes from syndromic tumors were compared against an internal mRNA expression database, MG-eDB1. The MG-eDB1 database comprises the expression profiles of a total of 278 various primary and metastatic brain tumors, including the syndromic tumors described in this study, processed and sequenced under the same experimental conditions [11, 14, 16, 17]. For the high-grade sarcoma clinical diagnostic workup, a methylation test was performed in St. Jude Children’s Research Hospital (Memphis, TN) on an Infinium MethylationEPIC BeadChip array (850 k array) targeting 862,927 CpG sites across the genome (Illumina Inc, San Diego, CA).

### Statistical analysis

Hierarchical clustering was performed as described [14], by using Instant Clue software [18]. Gene category overexpression median ranking was assessed by using non-parametric, two-tailed Wilcoxon matched-pairs signed rank test, as described [11]. Data were analyzed and plotted by using Microsoft Excel (Microsoft Corp., Redmond, WA), and GraphPad Prism (Version 9.3.1, GraphPad Software, La Jolla, CA).

## Results

### Novel syndromic malignancies may precede or follow MB

#### Patient 1

**Fig. 1:**
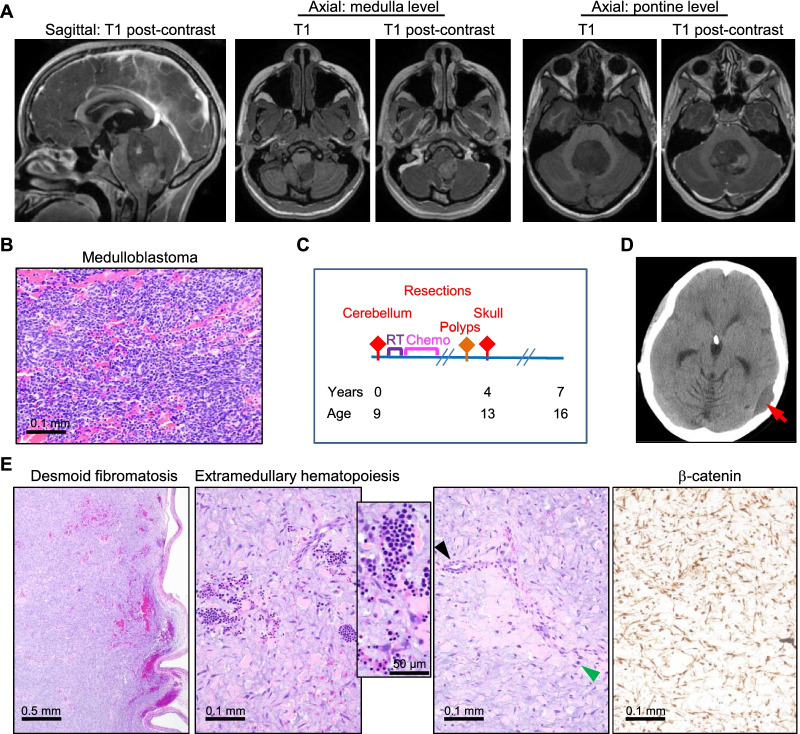
FAP syndromic desmoid fibromatosis arising 4 years after radiotherapy for MB. **A** MRI of a posterior fossa mass arising in floor of the 4th ventricle along the posterior aspect of the brainstem. **B** H&E shows the classic MB variant with abundant fresh hemorrhage. **C** Timeline of tumor progression and treatment. Rhomboid arrows indicate surgeries: intracranial in red, extracranial in orange; double slashes indicate interruption of the timeline for eventless periods; RT, radiotherapy; Chemo, chemotherapy. **D** CT of the epidural mass (red arrow) occurring 4 years post MB radiotherapy. **E** H&E of the epidural mass demonstrates an encapsulated hemorrhagic tumor composed of neoplastic fibroblastic cells embedded in a hyaline and myxoid ECM. Focal extramedullary hematopoiesis, showed magnified in inset, microvascular proliferation (black arrowhead) and mitotic activity (green arrowhead) are shown. β-catenin IHC shows diffuse and strong nuclear expression, indicating activation of the Wnt pathway

A 9-year-old Black/African-American female (F9) with family history of colorectal cancer (CRC) in mother and maternal grandmother, presented with a large posterior fossa mass arising in the floor of the 4^th^ ventricle and running along the posterior aspect of the brainstem, with patchy enhancement, especially in the caudal portion (Fig. 1A). Gross total resection was performed. Histologic examination showed a hemorrhagic embryonal cell tumor formed of sheets of relatively monomorphic small cells displaying nuclei with nucleoli and scattered mitotic figures, up to 15 per high-power field (Fig. 1B; Additional file 1: Fig. S2). Necrosis and vascular proliferation were absent. IHC performed at a different institution showed diffuse synaptophysin positivity, negative YAP1 and GAB1 staining, and β-catenin in cytoplasmic distribution. Of note is that some MB-WNT cases may show only focal nuclear β-catenin staining [1] that sometimes can be missed, underscoring the importance of NGS for correct classification. In the absence of NGS, the classic variant of MB-nonWNT/nonSHH was initially diagnosed in this case, and the standard regimen of craniospinal irradiation followed by chemotherapy (6 × 28-day cycles of vincristine, cyclophosphamide, cisplatin) was administered (Fig. 1C). Four years after MB resection, the patient underwent small and large intestine endoscopy that showed presence of tubular adenomas in the duodenum and jejunum (Fig. 1C). Approximately four years post-radiotherapy, an epidural mass was detected (Fig. 1D). Resection of the mass showed an encapsulated low-grade hemorrhagic neoplasm composed of cells with fibroblastic differentiation embedded in an abundant myxoid and hyaline extracellular matrix (ECM) (Fig. 1E). Extramedullary hematopoiesis was detected focally, in the absence of any hemoglobin abnormalities, a feature that may be present in some soft tissue neoplasms [19], and reported for the first time here in desmoid fibromatosis. Microvascular proliferation was also present focally, and neoplastic cells showed mitotic activity in a perivascular compartment (Fig. 1E, arrowheads). IHC with β-catenin antibody showed strong and diffuse nuclear staining (Fig. 1E). The diagnosis rendered was desmoid-type fibromatosis, a neoplasm that may occur in FAP [20]. Because of the association of MB and desmoid fibromatosis, an *APC* germline mutation was suspected.

#### Patient 2

**Fig. 2:**
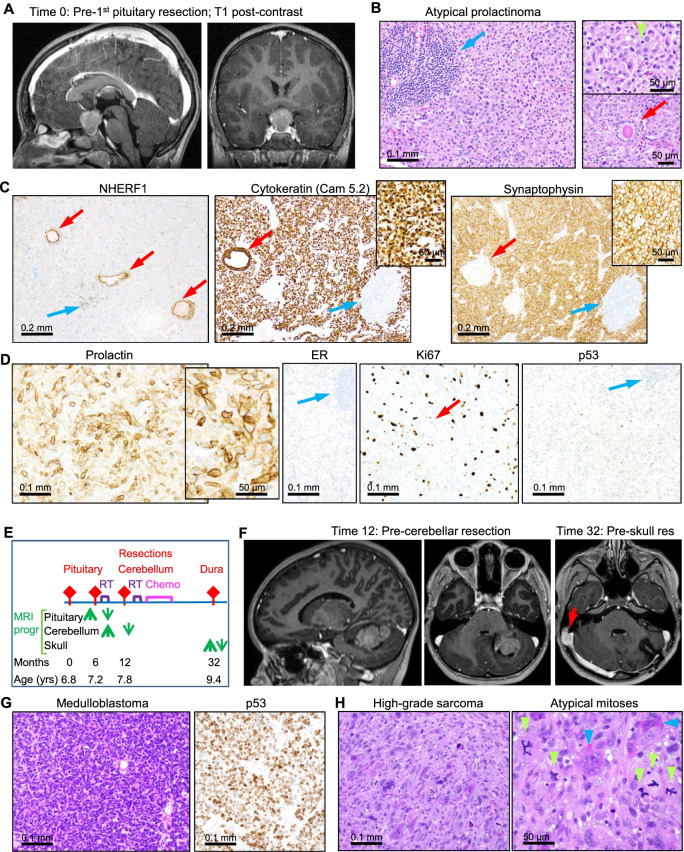
LFS MB and sarcoma progressing after radiotherapy for atypical prolactinoma. **A** MRI showing a macroadenoma compressing the optic chiasm and the floor of the 3rd ventricle. **B** H&E of the macroadenoma shows patchy lymphocytic inflammatory infiltrates (blue arrow), mitotic figures (green arrowhead) and glandular structures (red arrow). **C**, **D** IHC with indicated antibodies distinguishes the neoplastic cells from non-neoplastic inflammatory infiltrates (blue arrows) and glands (red arrows). Note NHERF1 labeling of glandular epithelial apical membrane and small T lymphocytes. The neoplastic cells show Cam 5.2 diffuse and strong cytoplasmic staining, synaptophysin and prolactin diffuse peripheral staining (insets with magnification), negative estrogen receptor (ER) staining, increased Ki-67 proliferation index and lack of p53 diffuse staining. **E** Timeline of tumor progression and treatment for the LFS M6 patient. Red rhomboid arrows indicate intracranial surgeries; green arrows (up – growth; down – decrease) indicate tumor progression observed on MRI (MRI progr); yrs, years; RT, radiotherapy (proton beam therapy); chemo, chemotherapy. **F** MRI of the two posterior fossa masses: left hemispheric cerebellar mass, and right dural mass (red arrow). **G** H&E and IHC with p53 antibody of the cerebellar mass show the large cell/anaplastic MB variant. **H** High-grade pleomorphic sarcoma shows numerous atypical mitotic figures (green arrowheads) and multinucleated osteoclast-like giant cells (blue arrows)

A 6-year-old White/Caucasian male (M6) with no significant family or past medical history presented with an intra- and suprasellar mass measuring 2.2 cm in diameter in the suprasellar component that compressed the optic chiasm and displaced the supraclinoid carotid arteries and the floor of the 3rd ventricle (Fig. 2A). The tumor was resected via transsphenoidal approach and pathology was consistent with a macroadenoma with patchy lymphoid inflammatory infiltrates (Fig. 2B). The tumor was composed of relatively monomorphic epithelial cells with abundant light eosinophilic (chromophobe) cytoplasm and large nuclei containing prominent nucleoli (Fig. 2B; Additional file 1: Fig. S3). Mitotic figures were conspicuous, in average 4 per 10 high power fields. Normal-appearing glandular structures were scattered within the tumor, and were lined by a monolayer of epithelial polarized cells labeled by NHERF1, a polarity marker structuring microvilli at the apical plasma membrane of epithelial cells [21, 22] (Fig. 2B, C). These glandular cells were also labeled by cytokeratin but not by synaptophysin (Fig. 2C), suggesting derivation from Rathke’s pouch. In contrast, tumor cells were diffusely and strongly labeled by both cytokeratin and synaptophysin. IHC with the six adenohypophysis hormones showed positivity only for prolactin, with a peripheral subplasmalemmal distribution (Fig. 2D; Additional file 1: Fig. S3). This peripheral pattern is novel, and is not characteristic of the two described histologic types of lactotroph adenoma/prolactinoma, sparsely or densely granulated, which show paranuclear or diffuse cytoplasmic staining, respectively [23]. The pre-operative levels of prolactin were only mildly elevated, at 50 ng/ml (normal levels 3.2–20 ng/ml). Electron microscopy was performed and peripherally placed small secretory vesicles were observed; however, “misplaced exocytosis”, an ultrastructural characteristic of prolactinoma, could not be visualized due to poor tissue preservation (not shown). Another unusual histologic finding was the lack of estrogen receptor labeling (Fig. 2D), a staining generally positive in prolactinoma [23]. The Ki-67 proliferation index was highly elevated, at 16.7%, and correlated with the mitotic count (Fig. 2D; Additional file 1: Fig. S3). IHC for p53 showed strong labeling of rare scattered nuclei, but the large majority of the cells were negative. Due to the atypical histologic features and high mitotic and Ki-67 proliferation indices, the tumor was diagnosed as atypical/high-risk prolactinoma, and frequent follow-up was recommended. Three months later, the tumor recurred and a second resection was performed (Fig. 2E). To prevent future recurrence, opposed lateral proton pencil beam radiation (54 CGE (Cobalt Gray Equivalent) in 30 fractions over 6 weeks) was administered resulting in efficient control of the pituitary tumor. Three months later, a new, rapidly growing, non-homogeneously contrast-enhancing, 4 cm cerebellar mass was detected and underwent gross total resection (Fig. 2E, F). Histologic examination showed a high-grade “blue” embryonal tumor exhibiting necrosis and vascular proliferation. The neoplastic cells showed hyperchromatic pleomorphic nuclei displaying molding and numerous mitotic figures, in average 22 per high-power field (Fig. 2G; Additional file 1: Fig. S2). IHC showed focal synaptophysin and GFAP, strong diffuse nuclear p53, patchy nuclear Olig2 expression, and a very high Ki-67 proliferation index, over 70%. The two IHC markers that are usually positive in the MB-SHH tumors, YAP and GAB1, showed an unusual pattern with patchy strong expression, including nuclear, for YAP, and weak and very focal expression for GAB1. The tumor was diagnosed as large cell/anaplastic histologic variant, suspicious for MB-SHH/TP53-mutant molecular subgroup, pending molecular characterization. Based on anaplastic histology, the tumor was treated as high-risk MB, with craniospinal irradiation (54 CGE in 30 fractions over 6 weeks) and concomitant weekly vincristine, followed by chemotherapy (6 × 28-day cycles of vincristine, cyclophosphamide, cisplatin). The patient was tumor-free for 10 months after therapy completion, being treated only for panhypopituitarism, when he developed a third, right posterior fossa, dural, bone-lytic mass (Fig. 2E, F). Histologic examination showed a high-grade pleomorphic sarcoma with very high mitotic index (30 mitoses per 10 high-power fields) and numerous atypical mitotic figures (Fig. 2H), suggestive for a radiation-induced sarcoma. IHC showed positive staining in subsets of neoplastic cells for h-caldesmon and CD163, weak for SMA, and strong labeling of multinucleated osteoclast-like giant cells by CD68 (Additional file 1: Fig. S4); negative stains included desmin, myogenin, S100 and EMA. The differential diagnosis of pleomorphic leiomyosarcoma versus undifferentiated pleomorphic sarcoma was formulated for this high-grade sarcoma, and a methylation array test performed for further tumor classification was non-contributory.

### Syndromic neoplastic associations revealed by genomic analysis

**Fig. 3:**
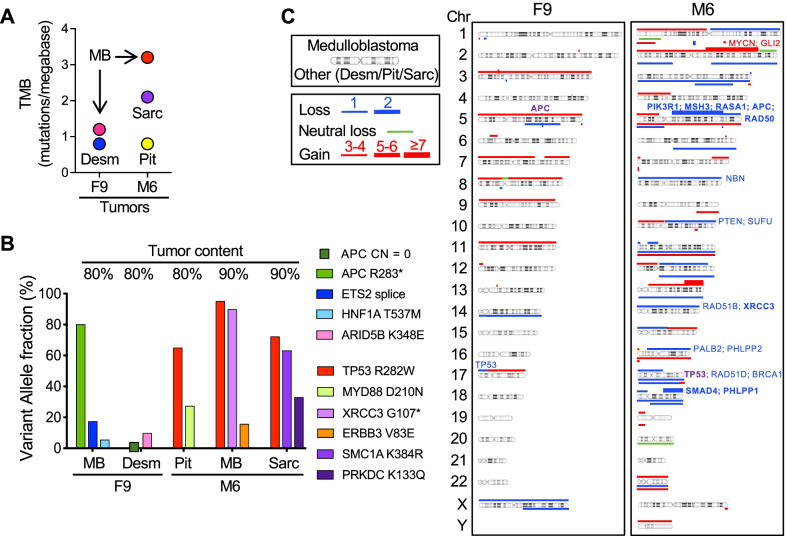
Genomic profiling of MBs and associated syndromic neoplasms. **A** TMB dot-plot showing very low mutation burden in the FAP-associated tumors from patient F9, in contrast to post-radiation tumors from patient M6. The tumor corresponding to each TMB value represented by a colored dot is indicated below the dot, except for the MB values that are indicated with arrows. Pit, pituitary adenoma/prolactinoma; Desm, desmoid fibromatosis; Sarc, high-grade sarcoma. **B** Graphic representation of the indicated genomic mutations showing their variant allele fraction. The *APC* homozygous loss (CN = 0) in patient F9 desmoid fibromatosis is indicated by a square corresponding to the value 0. **C** CN analysis shows above and below the chromosomes the profiles for MBs and associated syndromic neoplasms, respectively. For M6, the high-grade sarcoma profile is below the pituitary adenoma profile. Selected genes with expression change or pathogenic mutation are indicated: bold, double-hit; purple, syndromic genes (*TP53*, *APC*); red, oncogenes; blue, tumor suppressor genes

NGS was carried out for all five tumors. The TMB was very low in both F9 neoplasms and in the M6 pituitary tumor, but higher in the M6 MB and sarcoma occurring post radiotherapy (Fig. 3A). Consistent with the suspicion for syndromic disease, germline testing showed *APC* exon 5–15 deletion in patient F9. The pathogenic *APC* p.R283* truncating mutation was detected as second somatic hit in MB, establishing it as FAP-associated MB-WNT, whereas the *APC* locus showed homozygous CN loss in the desmoid fibromatosis (Fig. 3B; Additional file 1: Table S1). Similarly, *TP53* p.R282W pathogenic mutation was detected in the patient M6 germline, and all the tumors from this patient carried the deletion of the normal allele as second somatic hit. The R282W mutation maps to p53 DNA-binding domain and is listed as germline predisposing mutation for LFS (ClinVar, 15 submitters), establishing M6 MB as syndromic MB-SHH/TP53-mutant.

Somatic mutations were relatively rare, consistent with the low TMB of the neoplasms (Fig. 3B; Additional file 1: Table S1). The somatic variants in the F9 neoplasms showed subclonal allele fraction. The MB contained likely pathogenic variants in *ETS2* and *HNF1A* transcription factors. An *ARID5B* p.K348E pathogenic variant was detected in the desmoid fibromatosis. *ARID5B* encodes a transcription co-activator that has been shown to induce smooth muscle cell differentiation and reduced cell proliferation [24], and its mutation may have contributed to the pathogenesis of the tumor.

A *MYD88* p.D210N variant of unknown significance (VUS) with LOH was detected in the M6 aggressive pituitary adenoma. *MYD88* encodes a cytosolic adaptor protein involved in the innate and adaptive immune responses downstream of Toll-like receptors [25], and the D210N mutation maps to the TIR (Toll/Interleukin-1 Receptor) domain. Although its role in the pituitary adenoma pathogenesis is speculative at this time, it may be related to the presence of inflammatory infiltrates in this tumor [26]. The M6 MB showed a clonal *XRCC3* p.G107* truncating mutation with LOH, and a subclonal *ERBB3* p.V83E VUS in the extracellular domain of the receptor tyrosine kinase (RTK). *XRCC3* encodes a RAD51 paralog involved in DNA double-strand break (DSB) repair by homologous recombination (DSB-HR), and acting downstream of RAD51 recruitment to DNA sites of damage [27]. The M6 sarcoma showed mutations in two genes involved in DDR: *SMC1A* p.K384R, encoded on chromosome X and acting as an effector of the ATM/NBS1-dependent S-phase checkpoint [28], and *PRKDC* splice variant, encoding the catalytic subunit of DNA-PK involved in DNA DSB repair by non-homologous end joining (NHEJ) [29]. In-frame gene fusions of unknown significance were detected in the M6 sarcoma, including *COL1A2-EWSR1*, *FBXO25-SEPT14* and *CTBS-GNG5* (Additional file 1: Table S2).

The CN alterations showed relatively little overlap between the MBs (Fig. 3C). Important common alterations included loss of the 17p13.1 *TP53* site for all LFS-associated M6 tumors but also for the F9 MB, and loss of the 5q22.2 *APC* site for both FAP-syndromic F9 tumors but also for the M6 MB. The M6 tumors had partial overlap of chromosome 18 loss. The post-radiotherapy M6 MB and sarcoma had additional gain overlap on chromosomes 1p, 19p and 22. Whole chromosome 5 or 11 gain was common for F9 MB and M6 prolactinoma or sarcoma, respectively, and conversely, whole chromosome 14 loss, including the *XRCC3* site, was common for the M6 MB and F9 desmoid fibromatosis that occurred post-radiotherapy.

Whole or nearly whole chromosome gains predominated in the F9 MB, involving chromosomes 3, 5, 7, 8, 9, 11, 17, and only the X chromosome showed whole loss. In contrast, the F9 desmoid fibromatosis showed only scant CN alterations.

All LFS-associated M6 tumors showed numerous CN alterations, denoting chromosomal instability (Fig. 3C). The pituitary tumor contained whole chromosome 3, 5, 11, 16, 17, 18, 20 and 22 alterations, with losses slightly predominant over gains. Consistent with the marked chromosomal instability previously reported for the MB-SHH/TP53-mutant subgroup [9], the M6 MB showed relatively large homozygous losses on chromosomes 5q and 18q resulting in deletion of several tumor suppressor genes, including *PIK3R1*, *MSH3*, *RASA1*, *APC*, *RAD50*, *SMAD4* and *PHLPP1*. All these losses were accompanied by RNA expression decrease. Additional heterozygous losses of *NBN* on chromosome 8, *PTEN* and *SUFU* on chromosome 10, *RAD51B* on chromosome 14, *PALB2* and *PHLPP2* on chromosome 16, and *RAD51D*, *CDK12* and *BRCA1* on chromosome 17 were also accompanied by expression decrease. Overall, the CN losses impacted mostly the DSB-HR repair mechanism through alterations in *RAD50*, *NBN*, *BRCA1, PALB2*, *RAD51B*, *RAD51D*, *XRCC3*, and possibly *PHLPP1/2* [30–32]. Several tumor suppressors of growth signaling pathways were also disabled. *PTEN* and *PHLPP1/2* are known to suppress the PI3K/AKT signaling, and *PHLPP1/2* also the NF-*k*B pathway [33, 34], and *PIK3R1* and *RASA1* losses have been shown to activate the RAS-ERK/MAPK pathway [35, 36]. Chromosomal gains impacted mostly the expression of *MYCN* and *GLI2*, and together with *SUFU* loss, most likely elicited the activation of the Shh pathway. The M6 sarcoma showed extensive CN alterations on 12 autosomes, with gains on chromosomes 1p, 11, 16, 19p, 22, and losses of 2p-2q, 3q, 5p, 6q, 12q, 17 and 18q.

### General evaluation of the syndromic tumors by expression analysis

**Fig. 4:**
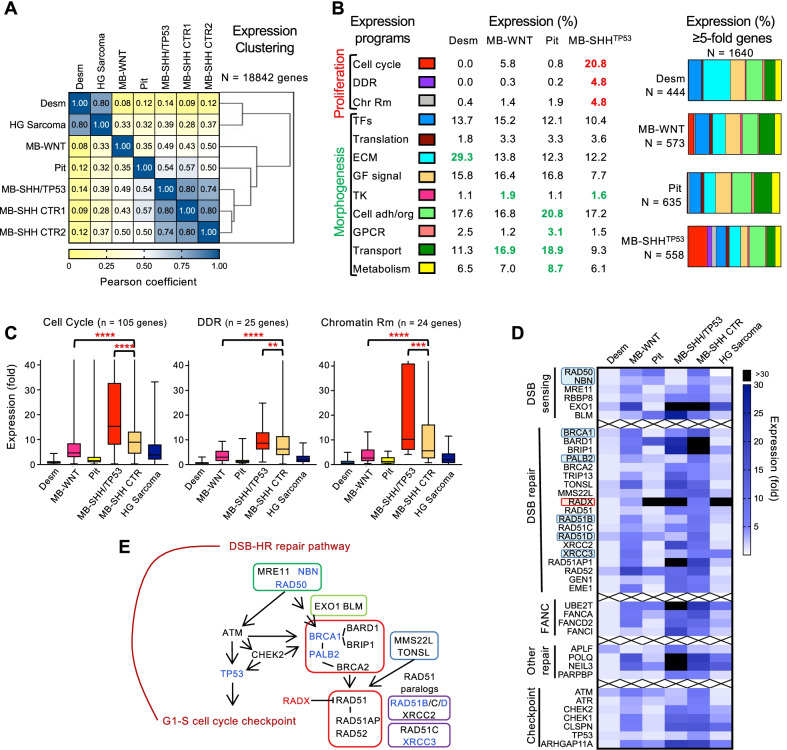
Proliferation expression programs in syndromic MB and associated neoplasms. **A** Hierarchical clustering of syndromic cases by multivariable Pearson correlation analysis. Note clustering of desmoid fibromatosis (Desm) with high-grade (HG) sarcoma, and of MB-SHH/TP53-mutant with two sporadic MB-SHH control (CTR) cases. The MB-WNT and atypical prolactinoma (Pit) show no clustering with the other tumors. **B** Expression programs classified in 12 color-coded functional categories for four of the syndromic tumors. Horizontal slice charts represent ≥ 5-fold overexpressed genes in each functional expression category. The respective functional category % is indicated in columns: red, predominant proliferative programs; green, predominant morphogenetic programs. The total and tumor-specific number of genes with ≥ 5-fold overexpression is indicated. Chr Rm, chromatin remodeling; TFs, transcription factors; GF, growth factor; TK, tyrosine kinase; Cell adh/org, adhesion/organization. **C** Overexpression ranking for the proliferation functional gene categories shown on top, and represented as box-and-whiskers plots of the overexpressed genes for the syndromic neoplasms and MB-SHH CTR. The box represents the median and quartiles, and the whiskers, the minimum and maximum values. P-values are indicated with red stars: ****p < 0.0001; ***p < 0.001; **p < 0.01. **D**, **E** Overexpression heatmap for DDR genes (**D**) and schematic representation of the different complexes involved in DSB-HR (DNA double-strand break repair by homologous recombination) (**E**). The genes labeled or boxed in blue show CN loss in the MB-SHH/TP53-mutant tumor from patient M6 (RAD50 and XRCC3 show double hit), and the gene boxed or indicated in red (*RADX*) shows specific massive overexpression in the M6 LFS tumors. In (E), the various protein complexes are boxed in: green for DSB sensing, red for main DSB repair components, blue for ancillary complex mediating assembly of RAD51 filaments on ssDNA, and purple for RAD51 paralogs

The current molecular classification of MBs is based on RNA expression clustering [1]. Transcriptome hierarchic clustering of the 5 syndromic cases and two sporadic control cases of MB-SHH TP53 wild-type classic variant with either homozygous *PTCH1* loss (MB-SHH CTR1 or CTR; see also Additional file 1: Fig. S2) or *SMO* mutation (MB-SHH CTR2) showed close grouping between M6 MB-SHH/TP53-mutant and the two MB-SHH control cases, and between the two soft tissue neoplasms, desmoid fibromatosis and high-grade sarcoma, regardless of their syndromic origin (Fig. 4A). The F9 MB-WNT and M6 pituitary adenoma were only distantly related to the MB-SHH subgroup cases, and the soft tissue tumor group was completely separated from the neuroendocrine tumors, as expected.

The tumor biological behavior was assessed as previously described [11] by examining the two large expression programs, proliferative and morphogenetic, composed of several functional categories (Fig. 4B). The expression in F9 desmoid fibromatosis was dominated by the ECM expression category, with high overexpression of genes encoding collagen matrix structural components and remodeling enzymes. Consistent with the tumor biology, the M6 pituitary adenoma expressed genes involved in G-protein couple receptor (GPCR) signaling, cell organization, transport, including cell trafficking, and metabolism. The M6 MB-SHH/TP53-mutant showed a strong proliferation program, correlating with the histologic aggressive features of this tumor, and also tyrosine kinase (TK) signaling. Only the latter was shared with the less histologically aggressive F9 MB-WNT that showed also an active transport program.

### Impairment of the DSB-HR repair pathway in the M6 MB-SHH/TP53-mutant

The examination of the proliferation expression programs showed significant differences between the syndromic MBs, correlating with the histology and prognostic classification (Fig. 4C). The cell cycle and chromatin remodeling programs were well developed in the M6 MB-SHH/TP53-mutant, whereas the DDR program appeared blunted. This was due to abolished expression in multiple effectors of the DSB-HR pathway (Fig. 4D, E). The MRN (MRE11-RAD50-NBS1) DNA damage sensing complex was disrupted by *RAD50* and *NBN* (encoding NBS1) expression loss secondary to CN loss (see Fig. 3C). Similarly, the BRCA1/BRCA2 complex and the RAD51 paralog complexes were disrupted by *BRCA1* and *PALB1*, and *RAD51B*, *RAD51D* and *XRCC3* expression loss, respectively. *BRCA1*, *RAD51D,* and additionally *XRCC2* were downregulated in the M6 high-grade sarcoma, whereas *XRCC3* was highly overexpressed. Interestingly, correlation between CN and expression was also apparent for the M6 pituitary adenoma, with *RAD50* overexpression correlating with CN gain, and *BRCA1* expression loss correlating with CN loss (Figs. 3C and 4D). In addition to MRN, BRCA1/2 and RAD51 paralog complexes, *ATM* also showed expression loss in the absence of CN variation in M6 MB-SHH/TP53-mutant, whereas *TP53* was inactivated in all M6 tumors. *RADX*, an antagonist of RAD51 [37], showed significantly higher overexpression in all M6 tumors compared to other tumors, in the absence of CN alterations. The Fanconi anemia complex involved in DNA cross-link repair and especially components of other DNA repair pathways showed high overexpression in M6 MB-SHH/TP53-mutant (Fig. 4D), possibly compensating for the DSB-HR repair deficiency.

### Tissue-dependent and independent morphogenetic signaling pathways in syndromic tumors

**Fig. 5:**
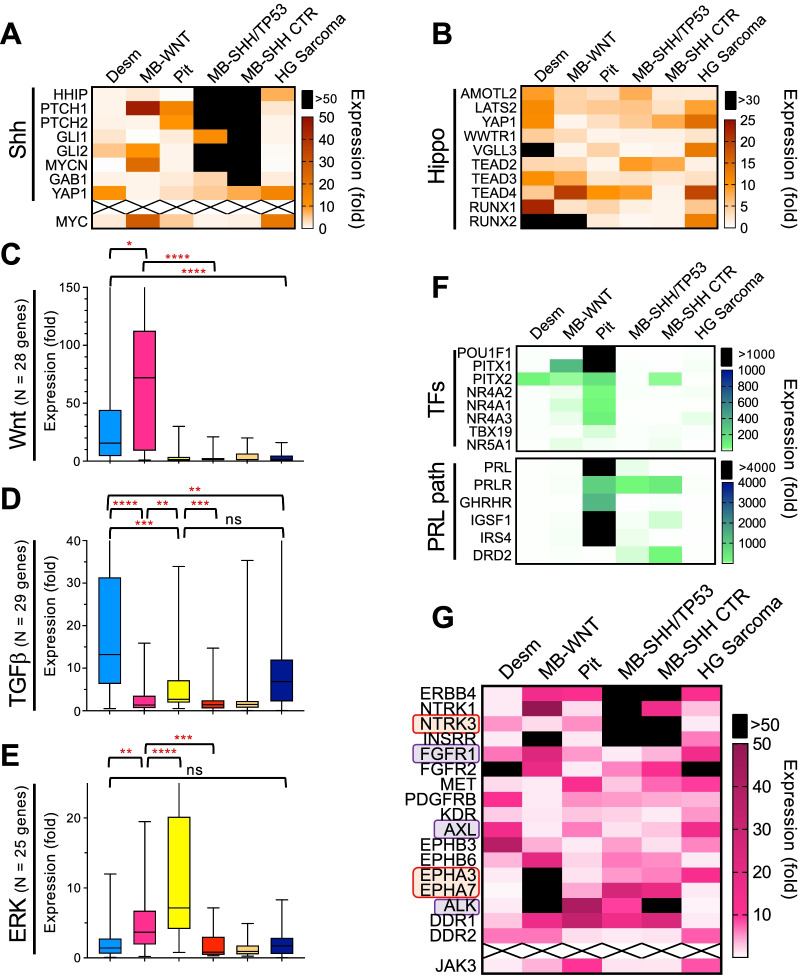
Morphogenetic pathway analysis in syndromic MB and associated neoplasms. **A**, **B** Heatmap of overexpressed genes from the Shh (selected genes) (**A**) and Hippo (**B**) pathways. *WWTR1* is also known as TAZ, and is the transcription factor that partners with YAP and TEAD-family or RUNX-family transcription factors for the activation of the Hippo pathway. VGLL3 is also a cofactor for the TEAD-family transcription factors. Note overexpression of these transcription factors in the FAP-associated desmoid fibromatosis (Desm), and MB-WNT, and also the Li-Fraumeni high-grade (HG) sarcoma. Pit, pituitary adenoma/prolactinoma. **C–E** The growth pathways Wnt-β-catenin (**C**), TGFβ (**D**) and ERK/MAPK (**E**) are represented by box-and-whiskers plots. Significant differences are shown with red asterisks: ****p < 0.0001; ***p < 0.001; **p < 0.01; *p < 0.05; ns, not significant. The tumor order is aligned to (A) labels. **F** Hormone specification pathways show the prolactin (PRL) secretory program massively activated in M6 atypical prolactinoma. **F** RTK fold-overexpression heatmap. The RTKs boxed in red or blue show overexpression levels significantly higher or in the 90^th^ percentile of expression values from the MG-eDB1 expression database (see Methods), respectively

The Shh pathway (Additional file 1: Fig. S1) was strongly activated in M6 MB-SHH/TP53-mutant, showing upregulation of a feedback inhibitor loop including *HHIP*, encoding a Shh inhibitor, and the *PTCH1-2* tumor suppressor genes [38] (Fig. 5A). On the other hand, *SUFU*, encoding the inhibitor of the GLI transcription factors was 2-to-3 fold downregulated in the MB-SHH/TP53-mutant, most likely due to CN loss (see Fig. 3C). *GLI2* and *MYCN* were also highly upregulated in MB-SHH/TP53-mutant, but also upregulated at lower levels in F9 MB-WNT, most likely as part of a cross-talk between Shh and Wnt pathways [39]. In comparison, *MYC* was upregulated in F9 MB-WNT that showed both *MYCN* and *MYC* high overexpression, and also in M6 high-grade sarcoma (Fig. 5A).

The downstream effectors GAB1 and YAP1 that are used as IHC markers for MB subgroup classification showed correlation with their IHC expression in both syndromic MBs (Fig. 5A and Additional file 1: Fig. S2). Strikingly, *YAP1* overexpression was much higher in the soft tissue neoplasms, suggesting activation of the Hippo pathway in these tumors, as YAP is the effector of the Hippo pathway in association with TAZ/TEAD transcription factors [40]. Other Hippo pathway effectors were overexpressed in both soft tissue tumors but also in the MBs in the absence of gene amplification, with massive upregulation of *RUNX1/2* transcription factors, as alternative partners for YAP [40, 41] (Fig. 5B; Additional file 1: Fig. S5).

The Wnt/β-catenin pathway (Additional file 1: Fig. S1) was massively and also specifically activated in both F9 FAP-associated tumors, regardless of their histologic origin (Fig. 5C). Notably, among the upregulated effectors was also *PDZRN3*, a Wnt pathway inhibitor [42] shown to promote vascular permeability and hemorrhage upon overexpression [43]. *PDZRN3* massive upregulation may have contributed and explains the hemorrhage observed in both F9 tumors (see Fig. 1). Interestingly, the *APC* homozygous loss in M6 MB-SHH/TP53-mutant did not result in Wnt/β-catenin pathway activation, perhaps due to late occurrence in the evolution of this tumor driven by the Shh pathway. Another morphogenetic pathway, the TGFβ/SMAD pathway was activated in the soft tissue tumors similarly to the Hippo pathway, and to a lesser extent in the M6 prolactinoma (Fig. 5D).

The ERK/MAPK pathway was highly activated in the M6 prolactinoma, and to a lesser but significant degree in F9 MB-WNT (Fig. 5E). In both cases, it involved the upregulation of the ERK negative feedback inhibitor loop previously described in glioblastoma [11], and also of downstream transcription factors *FOS*, *FOSB* and *JUNB*. In addition, *SHC1*, *SHC2* and *SHC3*, encoding the adaptor proteins activating Ras in complex with Grb2 and Sos, were highly and specifically overexpressed in the M6 prolactinoma.

### Detection of an autonomous prolactin autocrine loop in the M6 atypical prolactinoma

Within the morphogenetic expression programs, the pituitary hormone expression pathways were analyzed in order to correlate them with the prolactin-secreting histologic differentiation of the M6 pituitary adenoma. The transcription factors POU1F1 (also known as PIT1), TBX19 (also known as TPIT) and NR5A1 (also known as SF1) are responsible for the differentiation of adenohypophysis cells towards growth hormone (GH)/prolactin (PRL)/thyroid stimulating hormone, adenocorticorticotropic hormone or follicle-stimulating hormone/luteinizing hormone secretory programs, respectively [44]. Consistent with the histologic differentiation, the M6 pituitary adenoma showed massive upregulation of *POU1F1* and *PITX1*, high overexpression of *PITX2* and *NR4A1-3*, but minimal or absent expression of *TBX19* and *NR5A1*, respectively (Fig. 5F). PITX transcription factors have been shown to control the activity of the *PRL* promoter in concert with POU1F1 [45], and NR4A1 and especially NR4A2 transcription factors have been shown to impart selectivity for *PRL* gene expression in conjunction with POU1F1 [46]. Consequently, of the six adenohypophysis hormone genes, only *PRL* was massively overexpressed, in perfect correlation with the IHC results (Figs. 2D and 5F). Interestingly, the prolactin receptor gene, *PRLR*, was also massively overexpressed in M6 prolactinoma, but also in M6 MB-SHH/TP53-mutant. *GHRHR*, encoding the receptor for GH-releasing hormone was also upregulated to a lower level but specifically in the M6 prolactinoma. Two additional genes, *IGSF1* and *IRS4*, were massively and specifically overexpressed in the F6 prolactinoma. Inactivating mutations in both these genes have been implicated in the etiology of congenital central hypothyroidism, and the *IGSF1* deficiency that is more prevalent and better characterized has been shown to induce hypoprolactinemia and GH hypersecretion [47]. One of the unusual histologic features of the M6 prolactinoma was the absence of estrogen receptor expression by IHC. Examination of the expression for both *ESR1-2* genes and *PGR* showed very low values, in perfect correlation with the lack of staining for these receptors. Similarly, all 5 dopamine and somatostatin receptors displayed very low values, well below the threshold 500-count value, as shown for *DRD2*, encoding dopamine receptor 2 responsible for the inhibition of prolactin secretion, and showing CN loss (Fig. 5F).

### Therapeutic susceptibilities exposed by the RTK programs of syndromic neoplasms

RTK expression profiling showed upregulation of multiple RTKs in each tumor (Fig. 5G). Although no RTK showed gene amplification, three RTKs, *NTRK3* in the MBs-SHH, and *EPHA3* and *EPHA7* in the F9 MB-WNT, showed extremely high absolute levels of overexpression, two to four times above the level expressed by the highest expressor from the MG-eDB1 expression database (see Methods). Other RTKs, such as *FGFR1* and *AXL* in the soft tissue tumors, and *FGFR1* and *ALK* in MB-WNT, showed absolute values in the 90^th^ percentile of all tumors, revealing potential therapeutic susceptibilities. JAK3, a non-receptor TK intrinsic to the JAK/STAT growth pathway, showed approximately 10-fold overexpression in the M6 prolactinoma and high-grade sarcoma.

An RTK correlation matrix closely matched the results observed with whole transcriptomics clustering (Additional file 1: Fig. S6). The MBs-SHH had very close RTK programs, expressing high levels of *ERBB4*, *NTRK1/3*, *INSRR* and lower levels of the EPH family RTKs. The soft tissue neoplasms showed upregulation of RTKs related to fibroblast growth or ECM interaction and remodeling, such as *FGFR1/2*, *AXL*, *EPHB3* and *DDR2*. As noted, the F9 MB-WNT showed a robust RTK program (Figs. 4B, 5G), sharing with the MB-SHH cluster similar *NTRK1* and *INSRR* overexpression, and much higher EPH RTK overexpression, especially of *EPHA3/7*. In the M6 prolactinoma, *MET* was the only RTK with relative high overexpression, although the absolute levels were average in comparison to other CNS tumors, such as glioblastoma [14].

## Discussion

We showed here several tumor associations in pediatric patients with syndromic MB, of which two are novel: atypical prolactinoma and desmoid fibromatosis. In both patients, it was the presence of these preceding or subsequent neoplasms that triggered genomic testing for the MBs, establishing their syndromic nature and correct molecular subgroup classification. These observations emphasize the importance of NGS for the characterization of pediatric malignancies, in general. Indeed, is very important to recognize syndromic malignancies, as the management may be different than for sporadic tumors [2].

**Fig. 6:**
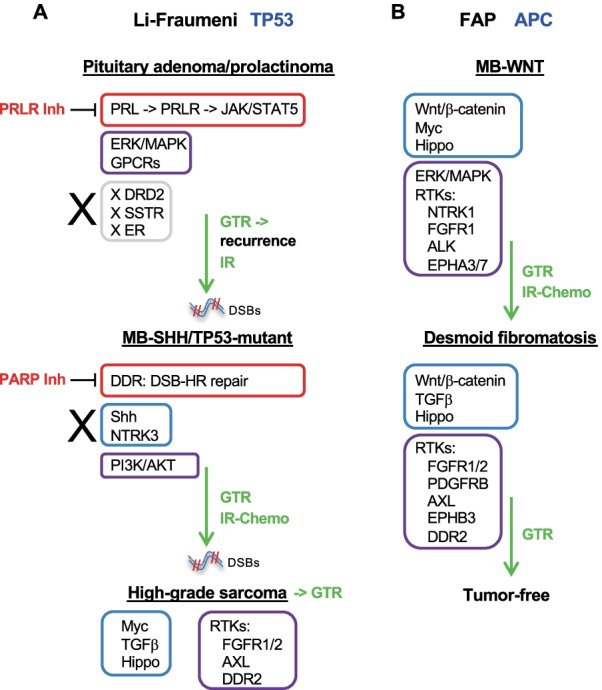
Pathway activation and therapeutic targeting in LFS (**A**) and FAP (**B**) syndromic tumors. The driver growth pathways are encircled in red if they are clinically targetable by pharmacological inhibitors (Inh); in blue, when either there are no clinically approved therapies or the therapeutic intervention is likely to be ineffective, the latter marked by an “X”; and in purple when potential inhibitors may be considered. Gray encircling and “X” marks absence of usually targetable pathways in the LFS atypical prolactinoma. The therapeutic interventions in the management of the tumors are indicated in green: GTR, gross total resection; IR, ionizing radiation; Chemo, chemotherapy

In this study, we found that the LFS-associated MB-SHH/TP53-mutant developed during and shortly after radiotherapy for atypical prolactinoma. The choice of proton beam therapy, a radiotherapy form that minimizes the risk for radiation-induced malignancies [48], effectively controlled the atypical prolactinoma. In one study, secondary tumor development has been described as a rare side effect of pituitary tumor radiotherapy, but with higher risk for younger ages [49]. However, studies addressing the rate and timeline of secondary malignancies in syndromic children with LFS or FAP are lacking. Although pre-existent transformation may have occurred in the cerebellum, the presence of *XRCC3* mutation in the MB-SHH/TP53-mutatant supports at least a major contributing effect of radiotherapy to the progression of the MB. In addition, the development of posterior fossa high-grade sarcoma after two different cranial radiotherapy regimens, further supports the increased sensitivity of pediatric LFS patients to ionizing radiation. Ionizing radiation induces DNA DSBs eliciting a strong DDR in radiated cells. The most likely oncogenic events in MB-SHH/TP53-mutant were the double “hits” in *XRCC3* and *TP53* that eliminated the accuracy of DNA repair and the DDR checkpoint, respectively, triggering marked chromosomal instability. The analysis of the DDR is instrumental for the choice of drug regimens, as radiotherapy and many drugs work by inducing DNA damage [29]. Through sensing mechanisms, targeted cells attempt to repair the damage by activating the various DNA repair mechanisms and the cell cycle checkpoints intended to allow time for repair. The gatekeeper p53 downstream of ATM activates cell cycle arrest and apoptotic mechanisms at a G1-S phase checkpoint. In LFS patients, due to p53 insufficiency, DNA errors may proceed unrepaired, leading eventually to cell transformation. The MBs developing spontaneously in LFS patients show dim prognosis after the usual therapy regimens [2]. Although the classification of MB is based on expression profiling, the analysis of the DDR pathways has not been addressed previously in MBs-SHH/TP53-mutant. Here, we showed that in the radiotherapy-induced MB-SHH/TP53-mutant, the DSB-HR repair mechanism was impaired at multiple levels, including the BRCA1/2 complex, whereas other repair pathways appeared intact (Fig. 6A). This context may be conducive to treatment with PARP inhibitors that have been shown to act by synthetic lethality in tumor cells with impaired BRCA1/2 complex [29]. This sensitivity may be additionally increased by the presence of *RADX* overexpression that has been shown to sensitize BRCA2-deficient cells to PARP inhibitors [37]. These findings also prompt the investigation of the DDR pathways in spontaneous LFS MBs or even in non-syndromic MBs-SHH/TP53-mutant in order to consider PARP inhibitors as potential therapy for this very aggressive subgroup of MBs.

The presence of multiple and histologically unrelated tumors in the same patient initiated by the same germline mutation conferred a unique opportunity for the assessment of signaling pathways and identification of lineage-dependent or -independent tumor markers and therapeutic susceptibilities. For the LFS tumors, the Shh pathway was activated only in the MB-SHH/TP53-mutant but not in the prolactinoma or high-grade sarcoma, suggesting that the activation of this pathway is tissue/progenitor cell-dependent (Fig. 6A). This interpretation is in concordance with the general gatekeeper role of p53 that, upon inactivation, would allow amplification of the tissue-specific proliferative signaling program once an oncogenic event occurred. In contrast, the *APC* germline alteration in the FAP tumors highly and specifically activated the Wnt pathway in both unrelated tumors, most likely by triggering additional oncogenic events in progenitor cells already depending for survival on the Wnt pathway (Fig. 6B). Unlike pituitary adenoma which is a new addition on the list of LFS neoplasms, desmoid fibromatosis has long been recognized as a FAP-associated malignancy [20]. However, the location, histologic appearance, and occurrence in the radiotherapy field of the prior MB confers new clinical features to this entity, as well.

Interestingly, this FAP-related soft tissue neoplasm closely clustered with the high-grade sarcoma from the LFS patient arising also post radiotherapy, and shared upregulation of common morphogenetic pathways, such as Hippo and TGFβ, and a common RTK program involved in fibroblastic differentiation (Fig. 6). High-grade sarcomas are the index diagnosis of LFS [6]. They may also occur post-radiation in non-syndromic patients [50], and both pleomorphic leiomyosarcoma and undifferentiated pleomorphic sarcoma are known to occur in both settings. Correlation between the *TP53* mutation site and the histologic type of sarcoma has been shown, and although the R282 missense mutation, as in M6, correlates best with spontaneous occurrence of liposarcomas and osteosarcomas in LFS carriers [51], the M6 high-grade sarcoma does not correspond to either of these types and matches better the post-radiotherapy sarcoma types [50].

Discerning signaling survival dependencies is essential for tailoring the adequate treatment of different tumors. For example, 10–20% of sporadic prolactinomas are resistant to the conventional therapy with dopamine agonists but, to date, only non-mechanistic clinical-histologic data are assessed in an attempt to predict aggressive behavior [52]. The LFS-associated recurrent prolactinoma was predictably resistant to the usual management by dopamine agonists not only due to its large size, elevated Ki-67 proliferation index and clinical relapse, but also to lack of expression of dopaminergic receptors, including D2, encoded by the *DRD2* gene (Fig. 6A). In addition, the tumor would not be a candidate to somatostatin receptor inhibitors, as the somatostatin receptor expression was low, as well. However, the expression analysis showed high overexpression of *PRLR* and *GHRHR*, both of these receptors representing known targets for chemotherapy in cancer [53, 54]. Prolactin binds PRLR that in turn recruits and activates the JAK-STAT5 pathway, inducing a proliferating response through cyclin D1 transcription [55]. Accordingly, *JAK3* and *CCND1* showed high expression levels in the M6 prolactinoma, indicating the existence of a proliferating autocrine loop in this tumor.

RTKs usually represent good targets for therapy and almost all have clinically-approved drugs. However, not all RTKs promote MB cell proliferation. For example, NTRK3 overexpression has been shown to induce apoptosis and tumor shrinkage in MB mouse models [56], and its inhibition in MBs-SHH may lead to tumor progression by increasing tumor cell survival. ALK overexpression by IHC detection has been recently proposed as marker for the MB-WNT subgroup [57]. In agreement with these data, we also found that *ALK* overexpression in FAP-associated MB-WNT was in the 99^th^ percentile of the large brain tumor MG-eDB1 expression database (Fig. 6B). As MBs-WNT respond well to current therapy regimens, more research is necessary to evaluate ALK inhibition in de-escalating regimens or for the very rare MB-WNT relapsing cases. In contrast, *EPHA3* or *EPHA7* overexpression has not been reported in MB, and our findings open a new research avenue addressing their role, specificity and targetability. Despite a series of RTK upregulation, the F9 FAP-associated desmoid fibromatosis that received only gross total resection, did not recur three years post resection, emphasizing the importance of the surgical treatment for this neoplasm (Fig. 6B).

## Conclusion

In conclusion, we present the clinical evolution of two syndromic patients developing MB and novel associated malignancies, and the integrated genomic-transcriptomic analysis that reflects syndromic, morphogenetic and post-radiation cues and reveal new therapeutic susceptibilities.

## Supplementary Information

**Additional file 1: Fig. S1**. Wnt and Shh signaling pathways. **Figure S2**. MB histology. **Figure S3**. Prolactinoma histology. **Figure S4**. High-grade sarcoma histology. **Figure S5**. The Hippo pathway in syndromic neoplasms. **Figure S6**. RTK correlation matrix. **Table S1**. Mutations. **Table S2**. In-frame fusions in M6 high-grade sarcoma.

## Data Availability

The datasets supporting the conclusions of this article are included within the article and its additional files.

## Abbreviations

APC: Adenomatous polyposis coli
CN: Copy number
CT: Computed tomography
*CTNNB1*: Encodes β-catenin
DDR: DNA damage response
DSB: Double-strand break
ECM: Extracellular matrix
ERK/MAPK: Extracellular signal-regulated kinase/mitogen-activated protein kinase
FAP: Familial adenomatous polyposis
FFPE: Formalin-fixed paraffin-embedded
FGFR: Fibroblast growth factor receptor
GFAP: Glial fibrillary acidic protein
GH: Growth hormone
GPCR: G-protein couple receptor
H&E: Hematoxylin eosin
HG: High-grade
HR: Homologous recombination
IHC: Immunohistochemistry
LFS: Li-Fraumeni syndrome
LOH: Loss of heterozygosity
MB: Medulloblastoma
MB-WNT: MB Wnt subgroup
MB-SHH: MB Shh subgroup
MB-SHH/TP53-mutant: MB Shh and TP53 mutant subgroup
MRI: Magnetic resonance imaging
NGS: Next generation sequencing
NHEJ: Non-homologous end joining
PI3K: Phosphatidylinositol 3-OH kinase
PRLR: Prolactin receptor
RTK: Receptor tyrosine kinase
Shh: Sonic Hedgehog
TGFβ: Transforming growth factor β
TMB: Tumor mutation burden
YAP1: Yes-associated protein 1
WHO: World Health Organization
Wnt: Wingless and Int-1
