# Supplementary material for: Novel neoplasms associated with syndromic pediatric medulloblastoma: integrated pathway delineation for personalized therapy
Source: Cell Commun Signal. 2022 Aug 17;20:123. doi: 10.1186/s12964-022-00930-3 (PMC9382778; doi:10.1186/s12964-022-00930-3)
Supplement: Supplementary file 2 — Additional file 1: Fig. S1. Wnt and Shh signaling pathways. Figure S2. MB histology. Figure S3. Prolactinoma histology. Figure S4. High-grade sarcoma histology. Figure S5. The Hippo pathway in syndromic neoplasms. Figure S6. RTK correlation matrix. Table S1. Mutations. Table S2. In-frame fusions in M6 high-grade sarcoma. [file 12964_2022_930_MOESM2_ESM.pdf]

SUPPLEMENTAL FIGURES S1-S6

S1: Wnt and Shh signaling pathways.
S2: MB histology.
S3: Prolactinoma histology.
S4: High-grade sarcoma histology.
S5: The Hippo pathway in syndromic neoplasms.
S6: RTK correlation matrix.

SUPPLEMENTAL TABLE S1-S2

S1: Mutations.
S2: In-frame fusions M6 sarcoma

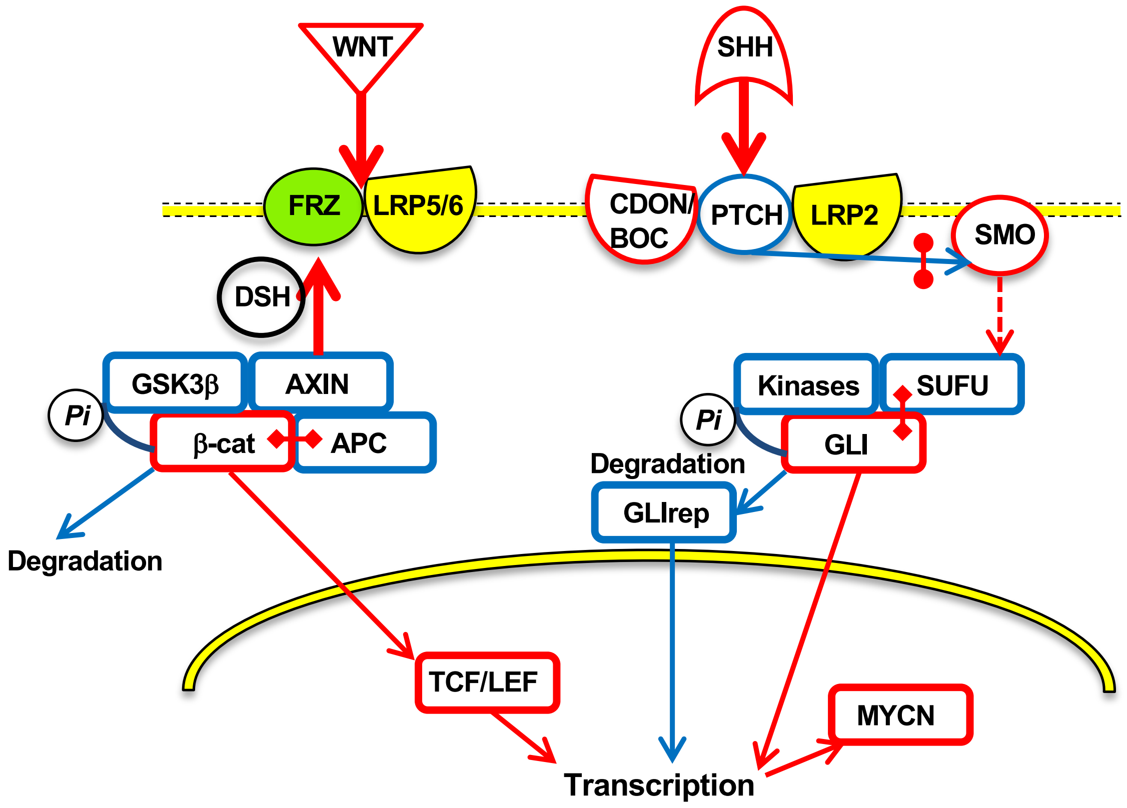

Supplemental Figure S1. Diagram of Wnt and Shh pathways. Schematic representations of the Wnt and Shh signal transduction pathways. The names of the genes encoding the proteins of the pathways are given, except for β-catenin (β-cat), encoded by *CTNNB1*, and GSK3β (Glycogen Synthase Kinase 3 Beta), encoded by *GSK3B*. Oncogenes and tumor suppressors are represented in red or blue shapes, respectively. Red and blue arrows denote actions carried out in activated or resting states, respectively; thick red arrows denote complex formation; double diamond lines denote disruption of complexes; double circle line denotes interruption of suppressive action.

Both pathways start by binding of the ligand WNT (Wingless) or SHH (Sonic hedgehog) to a plasma membrane receptor-coreceptor complex FRZ (Frizzled)-LRP5/6 (Low-density lipoprotein receptor-related) or PTCH (Patched)-LRP2-CDON/BOC (Cell adhesion molecule-related down-regulated by oncogenes/Brother of CDON), respectively. Note also similar reliance in resting state of both pathways on the inactivation by proteolytic cleavage (degradation) of the main signal transducer to the nucleus - β-catenin and GLI (Glioma-associated oncogene) transcription factors - triggered by their phosphorylation (*Pi*) either by GSK3β alone or with other kinases, respectively.

Briefly, Wnt binding its receptor triggers the recruitment of DSH (Disheveled) and AXIN, dissociating thus the β-catenin degradation complex composed of APC (Adenomatous polyposis coli), AXIN and GSK3β, and releasing β-catenin to translocate to the nucleus and complex with the TCF/LEF (T cell factor/lymphoid enhancer factor) family transcription factors to activate gene transcription.

The signal transduction via the Shh pathways involves interaction with primary cilium components (not shown in this diagram) and the release of the inhibitory action of PTCH on SMO (Smoothened) upon ligand binding. Active SMO dissociates SUFU (Suppressor of fused homolog) from GLI factors, through an unknown mechanism (dotted arrow), releasing their phosphorylation and proteolytic cleavage to repressor forms (GLIrep), and enabling them to translocate to the nucleus and activate transcription. MYCN is one of the direct targets of GLI transcription factors.

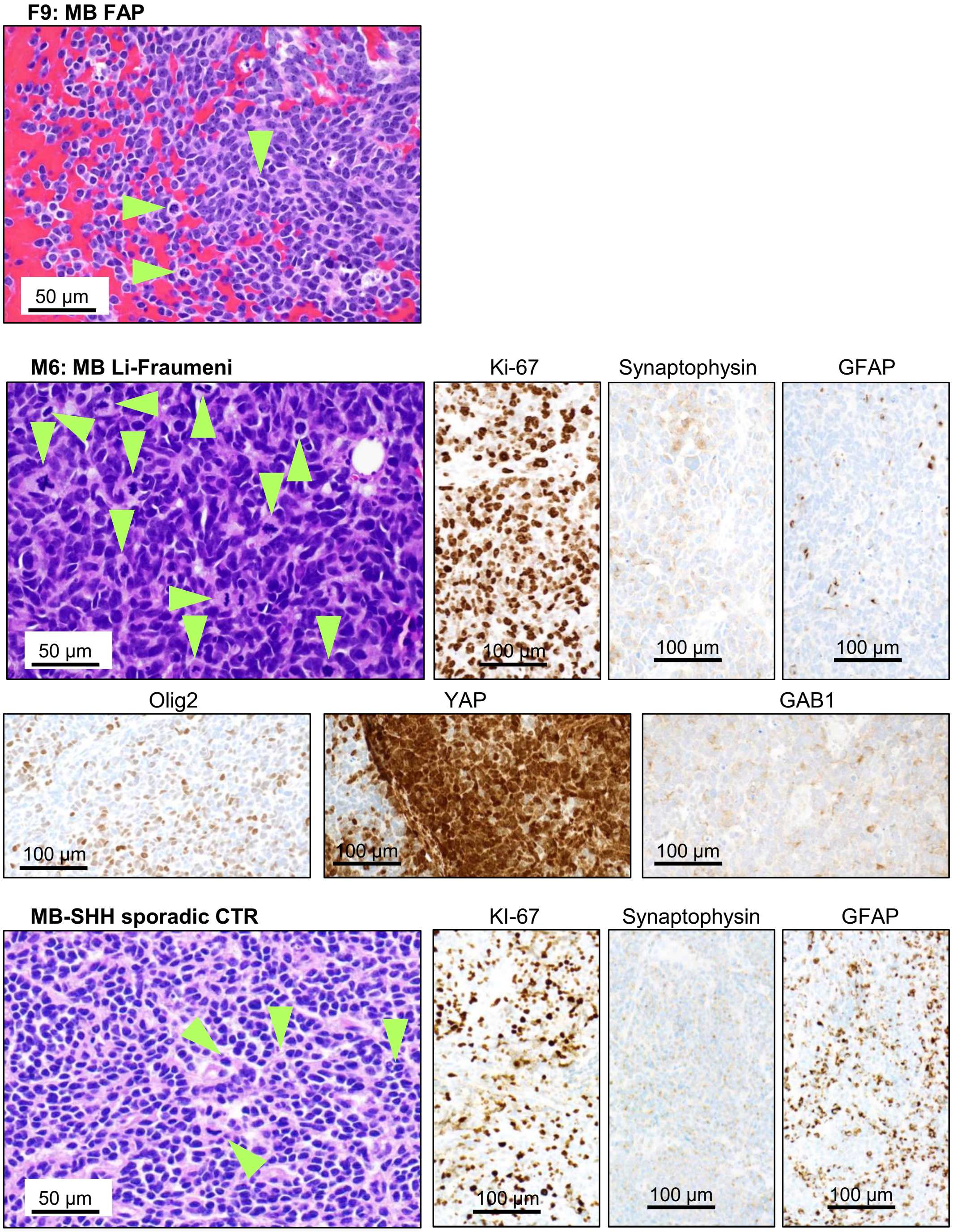

Supplemental Figure S2. **<u>Medulloblastoma (MB) histology.</u>** Hematoxylin-eosin (H&E) stain shows the classic variant with hemorrhage in the familial adenomatous polyposis (FAP)-associated MB WNT subgroup (MB-WNT) in patient F9, and the large cell/anaplastic variant in the Li-Fraumeni syndrome-associated MB SHH and *TP53* mutant subgroup (MB-SHH/TP53-mutant) in patient M6. A control (CTR) sporadic MB of SHH *TP53* wild-type subgroup (MB-SHH) classic variant is also shown for comparison. Mitotic figures are indicated by green arrowheads. Immunohistochemistry (IHC) with indicated antibodies is also presented for the MBs-SHH. Note in the M6 MB-SHH/TP53-mutant a field with patchy Olig2 nuclear positivity, a finding uncommon in MB, but suggestive of expression of stem cell markers of glial lineages. Although patchy YAP strong positivity is suggestive for MB-SHH subgroup, the nuclear reactivity is uncommon, and suggests activation of the Hippo pathway in this tumor (see also Fig. 5A-B). GAB1 low expression is similarly uncommon in the MB-SHH subgroup, and correlates with the mRNA expression from Fig. 5A.

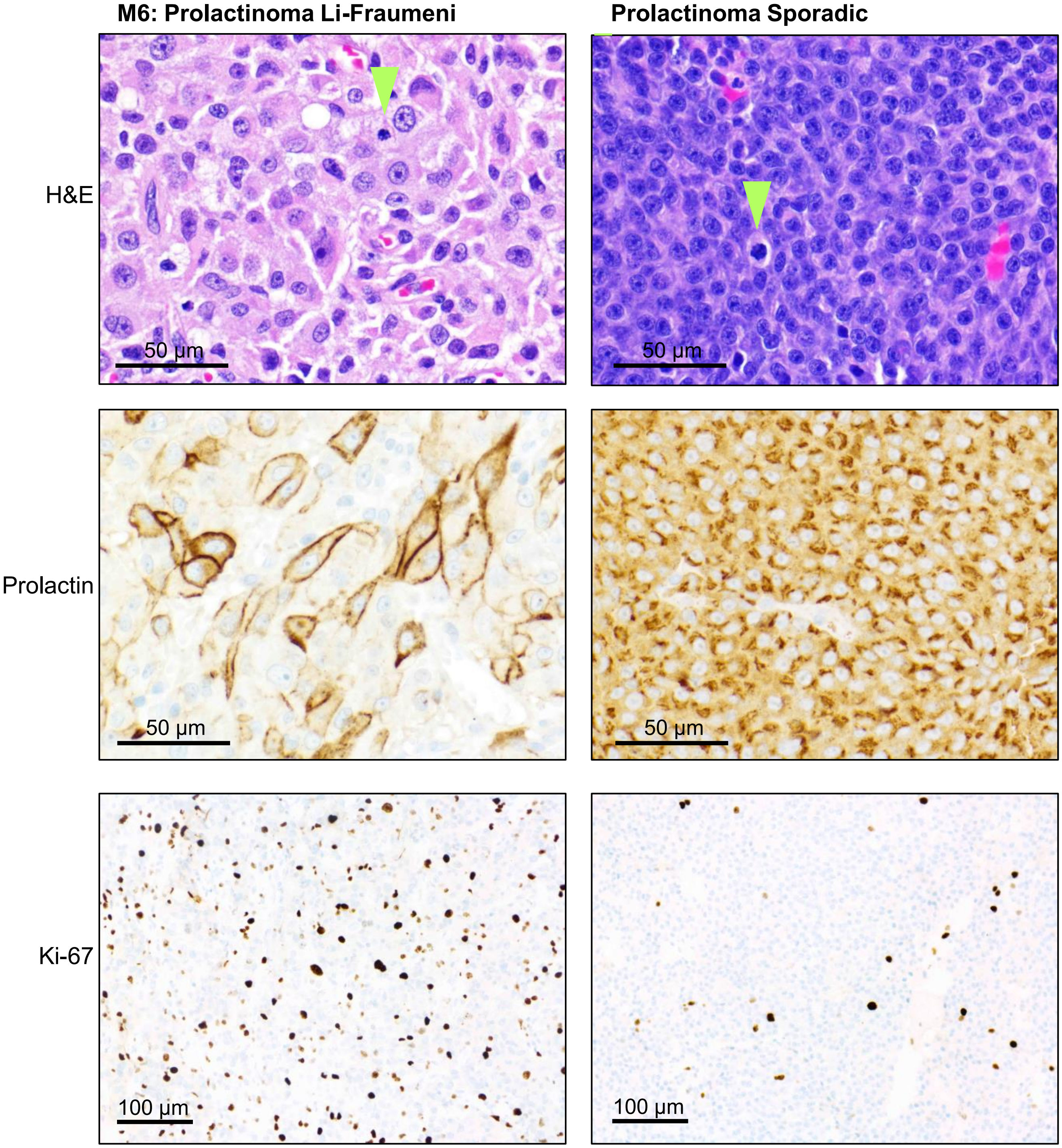

Supplemental Figure S3*.* **Prolactinoma histology: comparison between LFS and sporadic tumors.** H&E and IHC with prolactin and Ki-67 antibodies show major differences between the atypical prolactinoma occurring in LFS (patient M6) and a case of sporadic prolactinoma. Mitotic figures are indicated with green arrowheads. Note strong prolactin staining of the cell periphery in the M6 tumor in contrast to paranuclear staining in the sporadic sparsely granulated prolactinoma. Abbreviations as in Supplemental Figure S2.

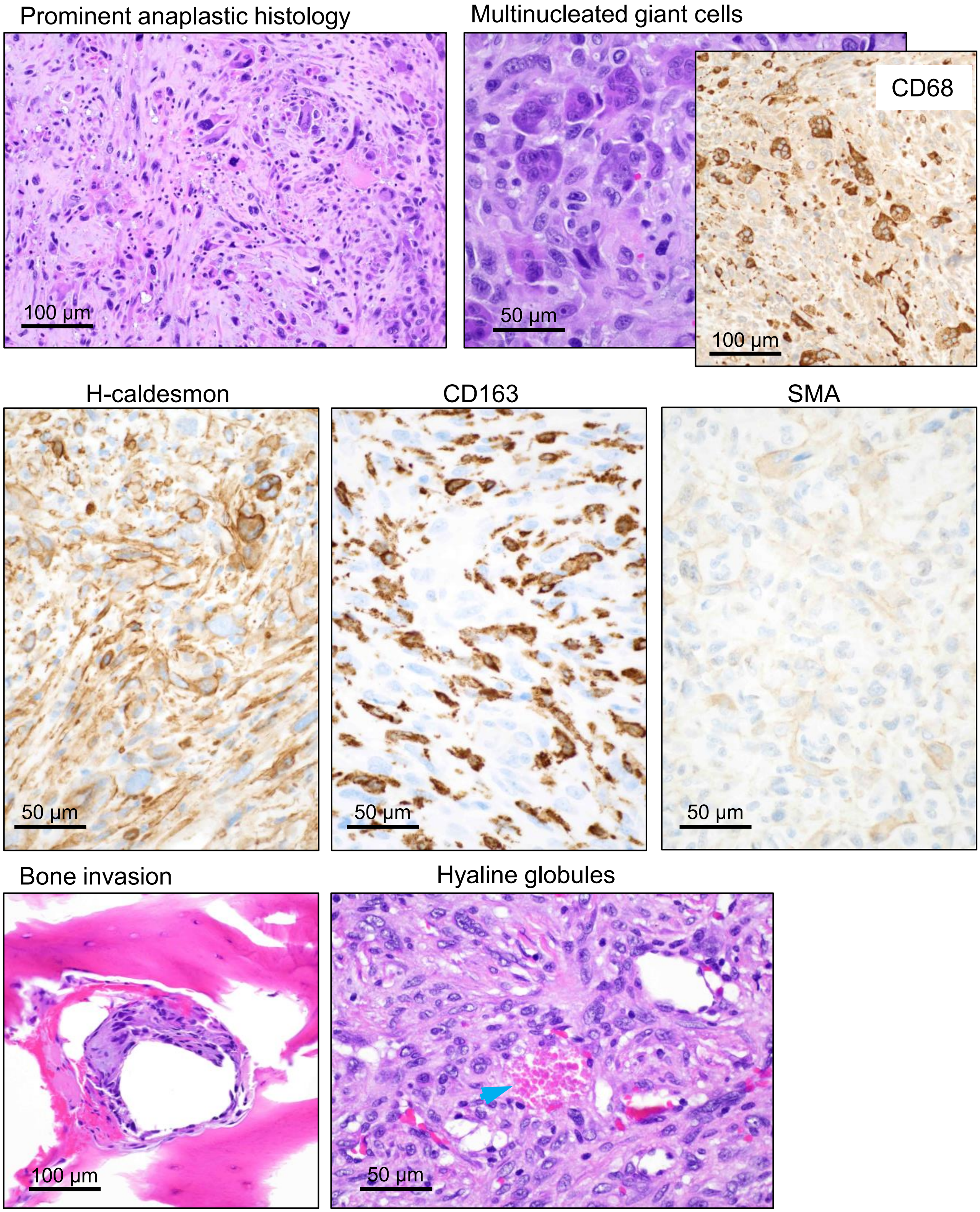

Supplemental Figure S4. <u>High-grade sarcoma histology.</u> Hematoxylin-eosin (H&E) shows various characteristics of the Li-Fraumeni associated post-radiation high-grade sarcoma, including prominent anaplastic histology, presence of multinucleated giant cells, bone invasion, and a focal cluster of hyaline globules (blue arrowhead). Immunohistochemistry with indicated antibodies is shown.

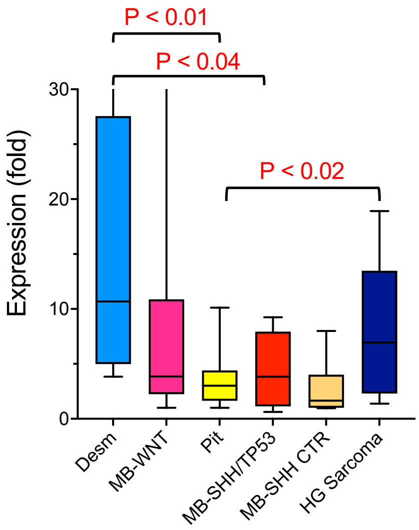

Supplemental Figure S5. **<u>The Hippo pathway in syndromic neoplasms.</u>** Box-and-whiskers plot of the overexpressed genes from Fig. 5B (n=10) from the Hippo pathway. Statistically significant differences are indicated. Desm, desmoid fibromatosis; Pit, pituitary adenoma/prolactinoma; HG, high-grade; MB, medulloblastoma ofs the following subgroups: WNT (MB-WNT), SHH and TP53 mutant (MB-SHH/TP53), SHH and TP53 wild-type (MB-SHH); CTR, control.

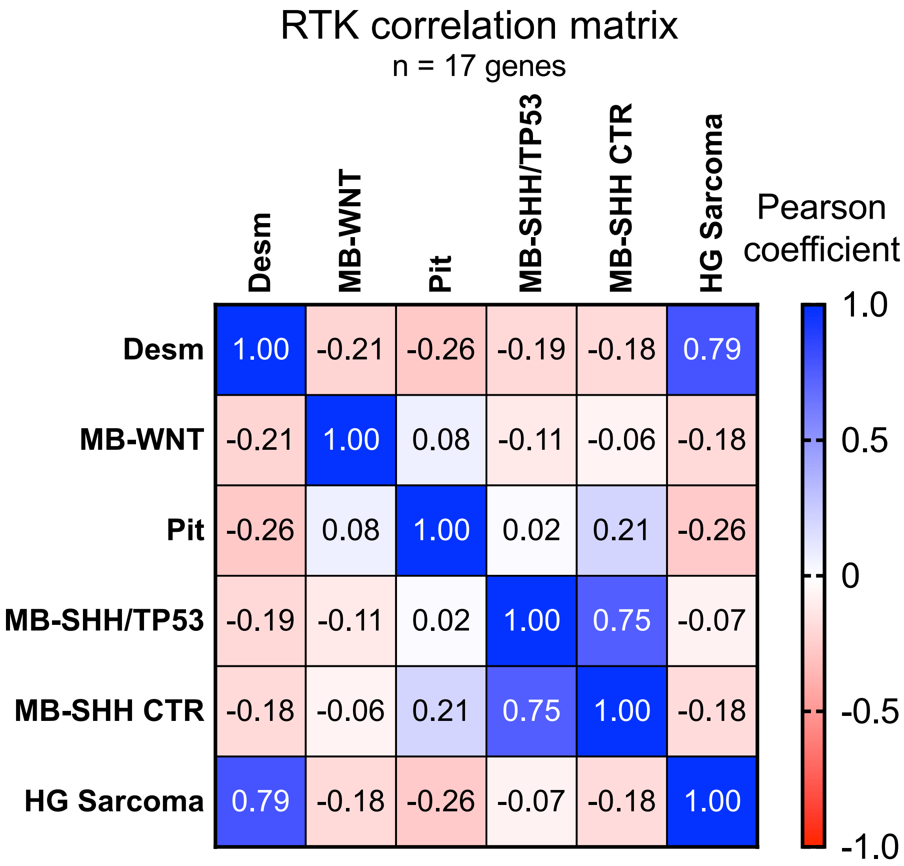

Supplemental Figure S6. **Receptor tyrosine kinase (RTK) correlation matrix.** Pearson correlation coefficient between each pair of tumors is shown. Note very similar RTK overexpression profiles between the soft tissue tumors desmoid fibromatosis (Desm) and high-grade (HG) sarcoma, regardless of syndromic origin, and the medulloblastomas Shh subgroup: MB-SHH/TP53-mutant and MB-SHH CTR (control). Note also divergent profiles of the remaining tumors, medulloblastoma Wnt subgroup (MB-WNT) and atypical pituitary adenoma/prolactinoma (Pit).

Supplemental Table S1. **Mutations**

Specimen	Gene	Mutation	Amino acid	Effect	LOH	Other	NM
**Patient F9**							
Blood	APC	exon 5-15		Deletion		Germline	_000038
MB-WNT	APC	exon 5-15		Deletion		Germline	_000038
	APC	c.847C>T	R283*	Nonsense	Yes	Somatic	_000038
	ETS2	c.421-4delT		Splice	No	Somatic	_001256295
	HNF1A	c.1610C>T	T537M	Missense	No	Somatic	_000545
Desmoid fibromatosis	APC	exon 5-15		Homozygous loss	Yes	Germline	_000038
	ARID5B	c.1042A>G	K348E	Missense	No	Somatic	_032199
**Patient M6**							
Blood	TP53	c.844C>T	R282W	Missense		Germline	_000546
Atypical prolactinoma	TP53	c.844C>T	R282W	Missense	Yes	Germline	_000546
	MYD88	c.628G>A	D210N	Missense	Yes	Somatic	_002468
MB-SHH/TP53	TP53	c.844C>T	R282W	Missense	Yes	Germline	_000546
	XRCC3	c.319G>T	G107*	Nonsense	Yes	Somatic	_005432
	ERBB3	c.248T>A	V83E	Missense	Yes	Somatic	_001982
High-grade sarcoma	TP53	c.844C>T	R282W	Missense	Yes	Germline	_000546
	SMC1A	c.1151A>G	K384R	Missense	Yes*	Somatic	_006306
	PRKDC	c.397A>C	K133Q	Splice	No	Somatic	_006904

* on chromosome X

LOH, loss of heterozygosity; NM_number, GenBank cDNA reference sequence; MB, medulloblastoma; MB-WNT, MB subgroup Wnt; MB-SHH/TP53, MB subgroup Shh and TP53-mutant

Supplemental Table S2. **In-frame fusions in patient M6 high-grade sarcoma**

5' Gene	Chr	Base pair	Transcript	Exon	3' Gene	Chr	Base pair	Transcript	Exon
COL1A2	7	94056948	ENST297268	49	EWSR1	22	29694723	ENST414183	15
FBXO25	8	385707	ENST276326	5	SEPT14	7	55863785	ENST388975	10
CTBS	1	85028940	ENST370630	6	GNG5	1	84967653	ENST370641	2

Chr, chromosome
